# Myocardial ischemia in patients with large prior infarction: Clinical decision making and review of literature

**DOI:** 10.1016/j.radcr.2022.11.006

**Published:** 2022-11-25

**Authors:** Talal Asif, Rami Doukky

**Affiliations:** a Division of Cardiology, University Health Truman Medical Center, 2301 Holmes Street, Kansas City, MO 64108, USA; b Division of Cardiology, Cook County Health, 1969 W Ogden Ave, Chicago, IL 60612, USA; c Division of Cardiology, Rush University Medical Center, 1620 W Harrison St, Chicago, IL 60612, USA

**Keywords:** Multivessel coronary artery disease, Rubidium-82 positron emission tomography, Transient ischemic dilatation, Myocardial blood flow, Myocardial flow reserve

## Abstract

Myocardial perfusion imaging (MPI) with single photon emission computed tomography (SPECT) or positron emission tomography (PET) is a widely used technique for the evaluation of coronary artery disease (CAD). Interpreting physicians rely on regional variations in myocardial radiotracer uptake between rest and stress images to identify hemodynamically significant epicardial coronary artery stenosis. However, interpretation of MPI is very difficult in patients with large infarcts where there is no scintigraphically normal reference myocardium for comparison. In these patients, the stress and rest images appear similar due to balanced ischemia in the non-infarct territory. There are no clear guidelines on how to approach these cases. We present a case of MPI with a large right coronary artery territory (RCA) infarct where the left main (LM) coronary artery territory has no relative comparator and the images looked the same on stress and rest. However, the patient had multiple high-risk ancillary findings including electrocardiographic (ECG) changes with regadenoson, transient ischemic dilatation (TID), large severe inferior infarct, low myocardial blood flow (MBF) and myocardial flow reserve (MFR), but most notably increased right ventricular (RV) uptake on the stress images that was a subtle clue that we were dealing with LM equivalent in non-infarct zone. The coronary angiogram confirmed our findings. Through our case, we provide a comprehensive approach and review of literature on how to approach such challenging encounters.

## Introduction

Myocardial perfusion imaging (MPI) with single photon emission computed tomography (SPECT) or positron emission tomography (PET) is a widely used technique for the evaluation of suspected or known coronary artery disease (CAD) [1]. It is presumed that regional variations in myocardial radiotracer uptake are a consequence of significant epicardial coronary artery stenosis [2]. In MPI, images are normalized such that myocardial region with the highest radiotracer uptake is designated as the “normal” reference and perfusion in other myocardial regions is determined as a percentage of the maximum activity region [2]. A shortfall of this technique is that in patients with multivessel CAD, even though the reference segment may itself be under-perfused, it is nonetheless designated as “normal”, yielding minimal or no relative perfusion deficits [2]. This concept of balanced ischemia leading to false negative results is well recognized and applies to both SPECT and PET-MPI [1].

Interpretation of MPI becomes even more challenging in patients with large infarcts where there is less or no scintigraphically normal reference myocardium for comparison [3]. In these patients, it is conceivable that the stress and rest images may appear similar due to balanced ischemia in the non-infarct territory. In such cases alternative parameters need to be sought [4].

Here we present a case with MPI findings that are concerning for significant left main (LM) coronary artery stenosis in a patient with a known large right coronary artery (RCA) territory infarct. PET derived myocardial flow reserve (MFR) and ancillary high-risk features enabled accurate diagnosis and guided appropriate management.

## Case presentation

A 75-year-old male patient was referred by his primary care physician for rest/regadenoson stress Rb-82 PET-MPI as part of pre-operative evaluation prior to vascular surgical intervention for right lower extremity chronic limb ischemia. He has a past medical history of CAD, status post percutaneous coronary intervention to the distal RCA and proximal left anterior descending coronary artery (LAD) 6 months prior, ischemic cardiomyopathy with ejection fraction of 25%, hypertension, hyperlipidemia, type-2 diabetes mellitus, and peripheral arterial disease.

**Fig. 1:**
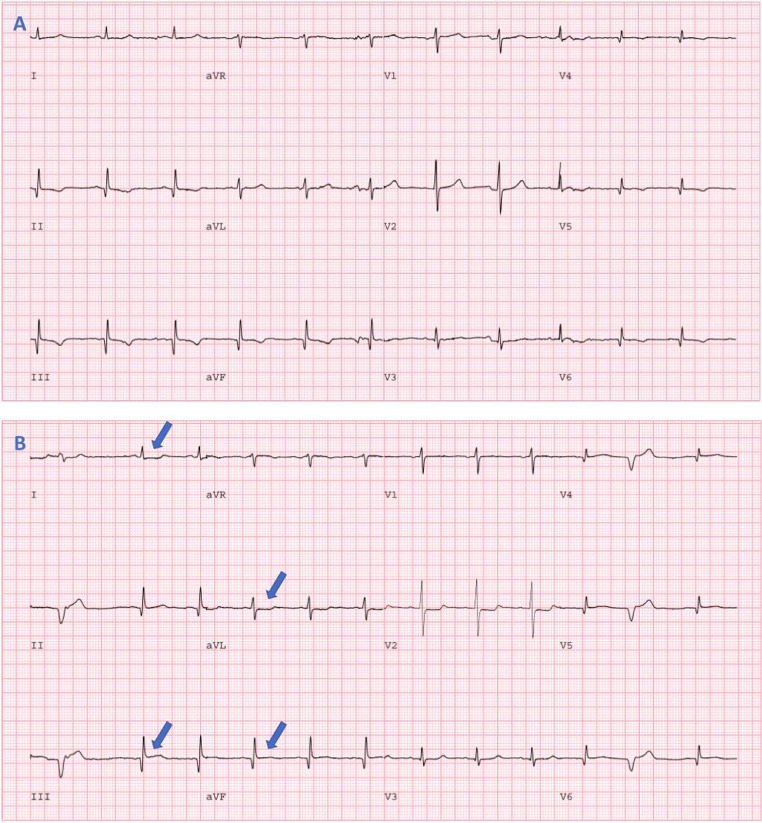
Rest and Stress ECG. (A) Resting ECG showing sinus bradycardia and inferior, lateral and possibly posterior myocardial infarction (inferior and lateral Q wave, poor R-wave progression in lateral leads, and tall R-wave in V2). (B) Stress ECG, following regadenoson injection, showing 1 mm ST-elevations in leads III and aVF with 0.5 mm horizontal ST depressions in leads I, aVL.

A standard dynamic rest and dynamic stress Rb-82 protocol with computed tomographic (CT) attenuation correction was employed. The patient was injected with 17.3 mCi of Rb-82, followed by image acquisition for 7 minutes. Pharmacologic stress with 0.4 mg intravenous regadenoson was then performed, followed by an injection of 17.3 mCi of Rb-82 and a 7-minute image acquisition. Patient's baseline electrocardiogram (ECG) showed sinus bradycardia with non-specific T-wave abnormalities (Fig. 1). With regadenoson stress, the patient had 1 mm ST-elevations in leads III and aVF with 0.5 mm horizontal ST-segment depressions in leads I and aVL (Fig. 1). The ECG changes persisted for 8 minutes into recovery. He did not complain of chest pain or dyspnea.

**Fig. 2:**
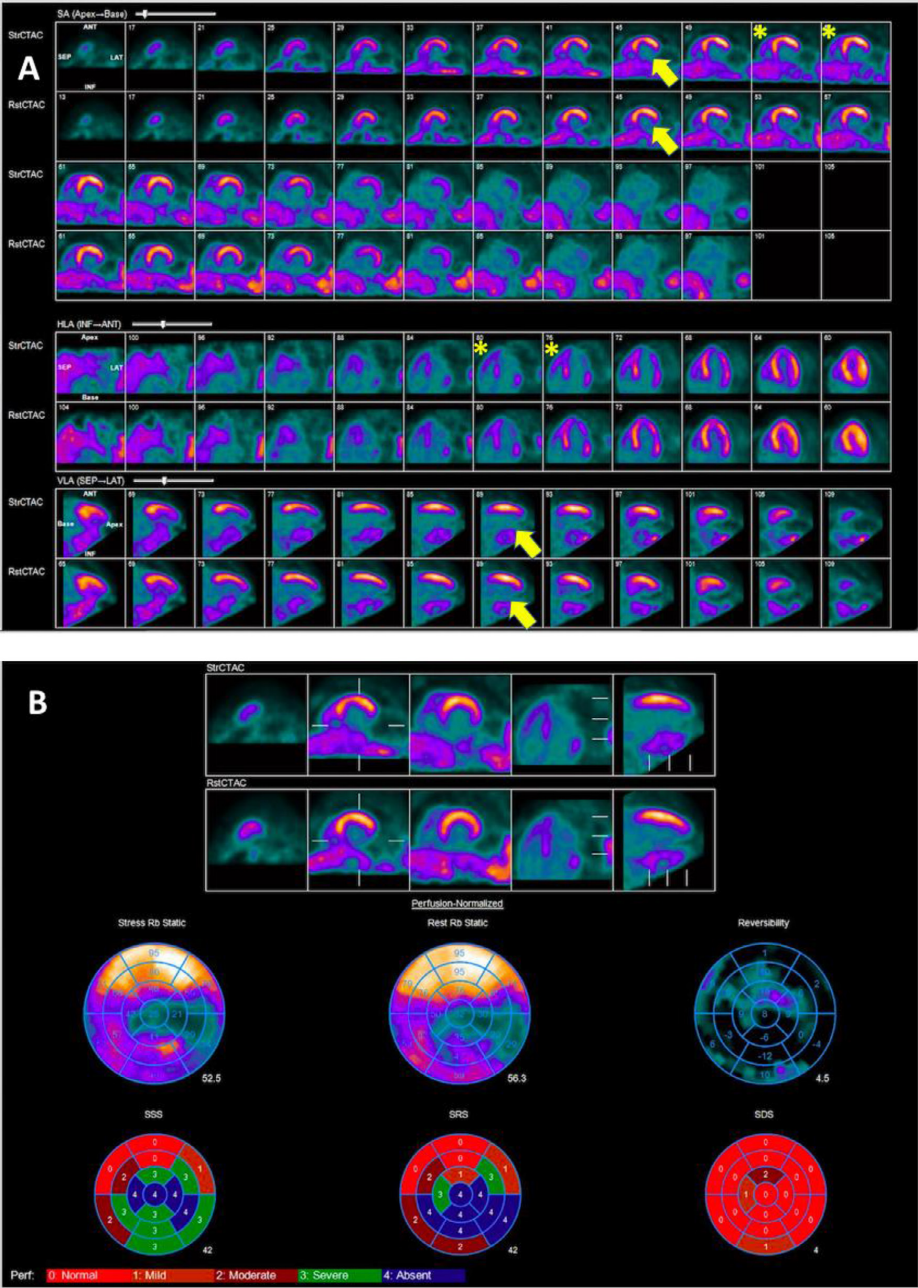
PET Myocardial Perfusion Imaging. (A) MPI shows large myocardial infarction in the RCA territory with minimal myocardial ischemia (yellow arrows) and increased RV uptake (asterisks). (B) Quantitative analysis showing predominantly fixed perfusion abnormality with trivial reversibility giving the perception of minimal ischemic burden.

**Fig. 3:**
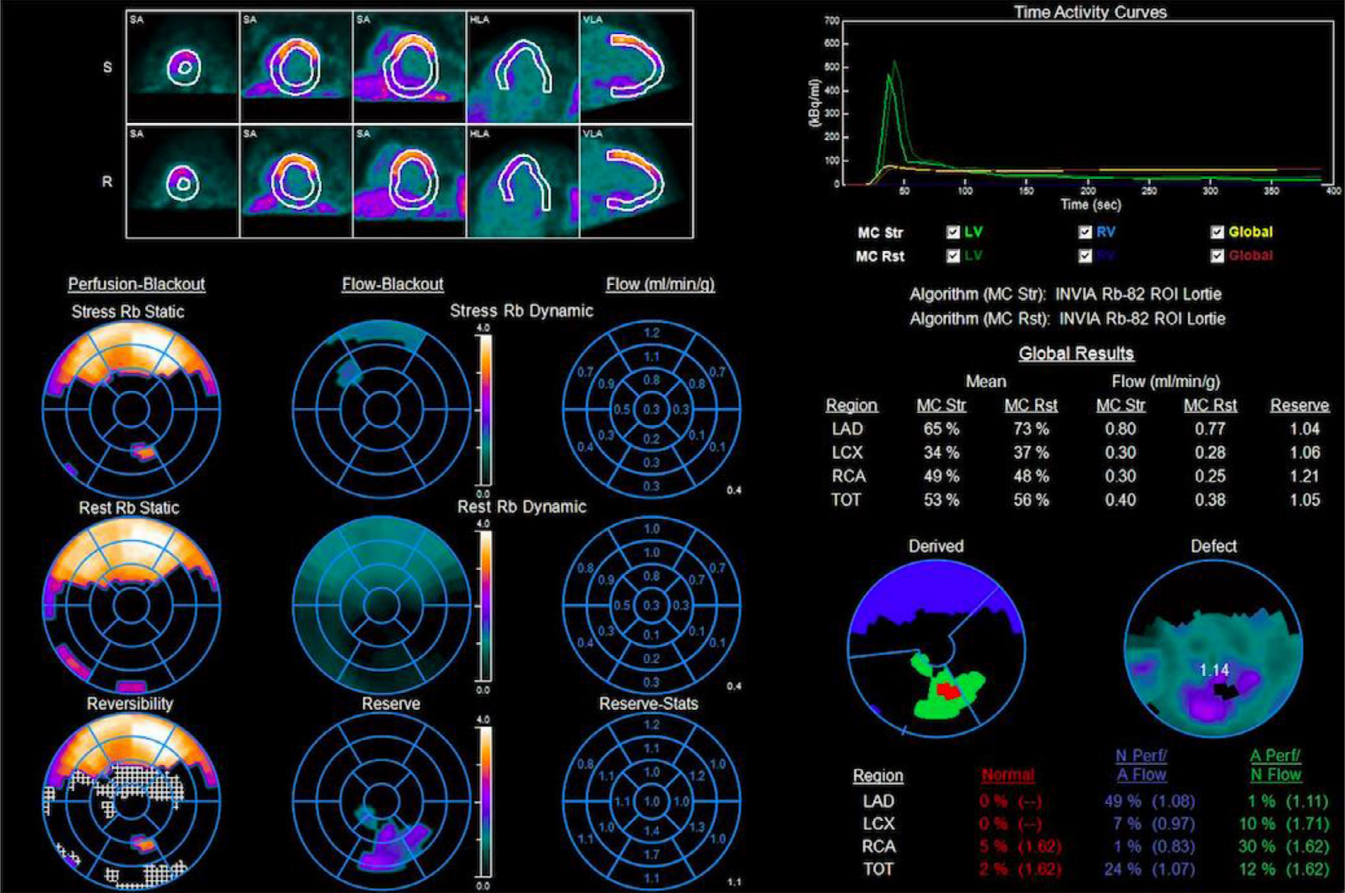
Myocardial Blood Flow. There is severe reduction in myocardial blood flow (MBF) at rest and stress with severely impaired myocardial flow reserve (MFR), especially in the territory of the left coronary artery.

MPI showed a large myocardial infarction in the distribution of RCA (Fig. 2). Since there was a large infarct in the distribution of the RCA, the myocardium supplied by the left coronary artery had no relative comparator. Therefore, visually, and quantitatively, the radiotracer uptake in the left coronary artery territory gave the impression of being similar on both rest and stress images (Fig. 2). Review of ancillary parameters revealed increased right ventricular (RV) uptake of the radiotracer at stress and a decline in left ventricular ejection fraction (LVEF) from 23% at rest to 21% at peak hyperemia. Additionally, the presence of transient ischemic dilatation (TID) of 1.28 further suggests multivessel coronary ischemia. Notably, there was a severe reduction in myocardial blood flow (MBF) at rest and stress with severely impaired myocardial flow reserve (MFR), including the territory of the left coronary artery (Fig. 3), which further suggested presence of high-risk multivessel CAD.

**Fig. 4:**
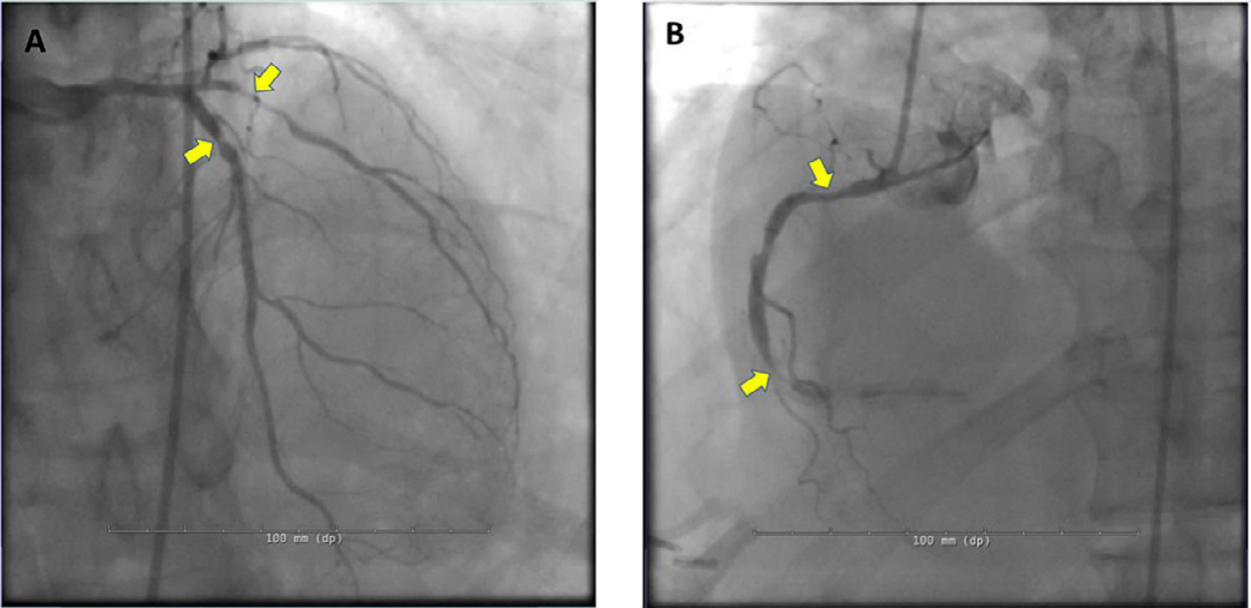
Coronary Angiography. (A) Severe in-stent restenosis of proximal LAD and proximal large obtuse marginal stenosis (yellow arrows). (B) 80% proximal and 99% distal RCA in-stent restenosis (yellow arrows).

Given the constellation of high-risk CAD findings on PET perfusion imaging and the presence of ischemic ECG changes with regadenoson, the patient was referred for invasive coronary angiography (ICA) that showed 80% in-stent restenosis of the proximal LAD, 90% tubular stenosis of proximal obtuse marginal branch, 80% proximal RCA stenosis, and 99% distal RCA in-stent restenosis (Fig. 4).

The patient underwent coronary artery bypass graft surgery during the same admission. Follow-up echocardiographic assessment showed improvement in his left ventricular ejection fraction to 35%.

## Discussion

The underestimation of the severity of CAD in patients with LM or multivessel CAD is a well-recognized shortcoming of relative SPECT and PET-MPI [5]. Studies have suggested that MPI alone may fail to spot multivessel CAD in up to 50% of cases [6]. The reasons postulated for the underestimation of CAD is a balanced reduction in myocardial perfusion which leads to the absence of a normal reference segment [7]. Traditionally, relative differences in radiotracer uptake in the left ventricular myocardium are used to quantitatively and qualitatively identify perfusion deficits [8]. In patients with balanced ischemia, there may be minimal heterogeneity in regional blood flow, giving the impression of a normal or near-normal scan [8]. Reversible perfusion defects may be even more challenging to identify in the presence of infarction in one or more coronary artery territories due to the absence of a scintigraphically normal reference segment [3]. In these situations, the addition of ancillary findings, such as ischemic electrocardiographic changes, increased right ventricular (RV) radiotracer uptake during stress, increased lung/heart tracer uptake ratio with thallium 201, transient ischemic dilation, wall motion abnormalities, and reduction in MBF and MFR can increase the sensitivity for high-risk coronary anatomy [6].

### Increased right ventricular uptake

Marked increase in relative RV radioisotope uptake with stress has been demonstrated as a marker for severe CAD [9,10]. In patients without significant CAD, the increase in LV radiotracer activity at stress is matched by a similar increase in RV radiotracer uptake such that the ratio of RV:LV uptake remains stable [9]. However, with severe CAD, the RV uptake may be normal but in the presence of severe LV hypoperfusion, the RV perfusion appears to be increased relative to the LV [9,10]. Though initially reported in exercise SPECT studies, increased RV uptake has been shown to have incremental diagnostic value in pharmacological PET-MPI [9,10]. It is important to be aware that pulmonary hypertension and RV hypertrophy may also demonstrate abnormally increased RV radiotracer uptake but patients with these conditions generally have increased RV:LV uptake ratios both at stress and rest rather than just at stress as observed in patients with severe CAD [9]. In our case, increased RV radiotracer uptake during stress was a clue that we were dealing with significant LM equivalent or multivessel CAD.

### Myocardial blood flow and myocardial flow reserve

Quantitative assessment of absolute MBF (mL/min/g) and MFR (peak hyperemic MBF / rest MBF) on PET-MPI has improved our ability to characterize the extent and severity of ischemia in multivessel CAD [11]. Studies have demonstrated that patients with obstructive 3-vessel CAD have globally reduced MFR, providing sensitive and incremental information to perfusion data obtained from MPI [1]. Furthermore, there is a step wise reduction in MBF and MFR with increasing severity of stenosis [11]. In general, a severe reduction in stress MBF of <1.5 mL/min/g and MFR of <1.5 can identify patients with multivessel CAD in the right clinical context [11]. Thus, MBF and MFR enabled us to meet additional diagnostic needs in patient care not met by MPI alone [12]. MFR provides an additional risk stratification tool and can lead to effective post-test risk reclassification in high-risk patients [11–13]. There is evidence that this improved risk stratification with MFR can lead to more selective downstream utilization of invasive diagnostic procedures and improved revascularization strategies [12,14]. Although the addition of MBF and MFR have improved our ability to recognize balanced ischemia, these parameters in isolation lack specificity for differentiation of left main/multivessel CAD from diffuse microvascular dysfunction [7]. This necessitates evaluation of coronary anatomy in high risk patients [7]. Nevertheless, in cases of severely reduced MFR, such as in our case, severe multivessel CAD should be strongly considered [1]. MFR should also be interpreted with caution in patients with large infarcts, as MFR may appear preserved due to severe decrease in resting blood flow associated with scar tissue [11]. As demonstrated by our case, stress MBF and MFR data should be used to complement other high-risk findings.

### Ischemic electrocardiographic changes

Vasodilator stress, principally using regadenoson, is the most common modality of stress MPI in the US [15,16]. Although majority of information is derived from MPI, it is important to review patient's ECG response to vasodilator stress since it can provide incremental diagnostic and prognostic information [15,17]. While ST-segment depression with regadenoson stress is uncommon, when present should be considered in overall decision-making [15]. It has been shown that regadenoson-induced ST-segment depression of ≥0.5 mm is associated with a higher prevalence of severe/extensive CAD [17] and higher incidence of major adverse cardiac events in patients with both normal and abnormal MPI [15]. Ischemic ECG changes with vasodilator stress are hypothesized to represent a surrogate for severe CAD due to coronary steal phenomenon that induces true myocardial ischemia [15]. In our patient, presence of regadenoson-induced ECG changes were an additional diagnostic clue for severe CAD.

### Transient ischemic dilatation

TID refers to enlargement of the LV cavity on post-stress MPI when compared to resting images [18]. Potential mechanisms for TID include ischemia induced LV dilatation, subendocardial ischemia with reduced subendocardial radiotracer uptake leading to an appearance of LV dilatation, and post-stress LV stunning. [18] TID is well validated as a marker for severe multivessel CAD and as a predictor of adverse cardiac outcomes in the presence of abnormal MPI [18,19]. In a meta-analysis, Alama et al. demonstrated that TID is a specific (pooled specificity of 88%) but not a sensitive (pooled sensitivity of 44%) marker of severe CAD [19]. The authors advocated that in the presence of TID and high-risk MPI findings, as in our patient, ICA should be strongly considered. [19] Some authors suggested that among patients with diabetes mellitus or known CAD, TID with otherwise normal MPI may be a marker of increased risk and may justify further cardiac work up [18,19]. As with SPECT, TID derived from PET-MPI provide similar diagnostic value [20]. TID in our patient was another hint that we were encountering severe/extensive CAD.

### Left ventricular ejection fraction reserve

LVEF reserve, defined as stress LVEF minus rest LVEF, has been shown to be a predictor of severe LM/3-vessel disease on pharmacologic PET imaging [21]. In patients undergoing Rb-82 PET-MPI, Dorbala et al. showed that a preserved LVEF reserve of ≥5% had a 97% negative predictive value for excluding severe LM or severe 3-vessel CAD [21]. Our patient demonstrated a reduction in LVEF during peak stress and therefore had abnormal LVEF reserve (-2%) which intensified our suspicion of severe LM or 3-vessel disease, warranting ICA. It is important to note that the LVEF reserve has not been shown to offer significant diagnostic and prognostic value in patients undergoing rest/regadenoson stress gated SPECT-MPI [22,23]. This is because Rb-82 PET-MPI enables measurement of LVEF at maximal hyperemia, unlike vasodilator SPECT where gated post-stress MPI are obtained approximately 45 minutes after stress [21].

### Regional myocardial dysfunction

Evaluation of myocardial wall motion and thickening on gated MPI can be performed with a reasonable degree of accuracy [24,25]. Post-stress wall motion abnormalities when present may be a marker of extensive CAD [26]. Kapetanopoulos et al. demonstrated that regional wall motion abnormalities (RWMA) on post-stress gated SPECT-MPI were an independent predictor of cardiac events [26]. The authors postulated that the mechanism of RWMA was post-ischemic stunning or severe subendocardial ischemia due to severe coronary stenosis [26,27]. Even though in our case RWMA were hard to identify due to the large underlying RCA infarct and globally reduced LV function, regional wall motion data may serve as an important non-perfusion finding for severe CAD.

## Conclusion

Severe left coronary circulation stenoses can be difficult to detect in the presence of large myocardial infarction in the RCA territory. Our case serves as a reminder to probe for high-risk ancillary parameters in patients with a high probability of severe CAD. PET-MPI is a better modality in high-risk patients with complex CAD due to the ability of MFR and LVEF reserve to provide strong additional prognostic and diagnostic data that SPECT cannot provide.

## Patient consent

Written informed consent for publication was obtained from the patient.
